# Enhancing two-stage object detection models via data-driven anchor box optimization in UAV-based maritime SAR

**DOI:** 10.1038/s41598-024-55570-z

**Published:** 2024-02-27

**Authors:** Beigeng Zhao, Rui Song

**Affiliations:** College of Public Security Information Technology and Intelligence, Criminal Investigation Police University of China, Shenyang, China

**Keywords:** Climate sciences, Environmental sciences, Hydrology, Natural hazards

## Abstract

The high-altitude imaging capabilities of Unmanned Aerial Vehicles (UAVs) offer an effective solution for maritime Search and Rescue (SAR) operations. In such missions, the accurate identification of boats, personnel, and objects within images is crucial. While object detection models trained on general image datasets can be directly applied to these tasks, their effectiveness is limited due to the unique challenges posed by the specific characteristics of maritime SAR scenarios. Addressing this challenge, our study leverages the large-scale benchmark dataset SeaDronesSee, specific to UAV-based maritime SAR, to analyze and explore the unique attributes of image data in this scenario. We identify the need for optimization in detecting specific categories of difficult-to-detect objects within this context. Building on this, an anchor box optimization strategy is proposed based on clustering analysis, aimed at enhancing the performance of the renowned two-stage object detection models in this specialized task. Experiments were conducted to validate the proposed anchor box optimization method and to explore the underlying reasons for its effectiveness. The experimental results show our optimization method achieved a 45.8% and a 10% increase in average precision over the default anchor box configurations of torchvision and the SeaDronesSee official sample code configuration respectively. This enhancement was particularly evident in the model’s significantly improved ability to detect swimmers, floaters, and life jackets on boats within the SeaDronesSee dataset’s SAR scenarios. The methods and findings of this study are anticipated to provide the UAV-based maritime SAR research community with valuable insights into data characteristics and model optimization, offering a meaningful reference for future research.

## Introduction

Remote sensing imagery^1,2^ refers to earth surface images captured from high altitudes by satellites, high-altitude cameras, or unmanned aerial vehicles (UAVs). The maturation and widespread application of UAV technology^3,4^ in recent years have significantly increased its adoption by a variety of enterprises and organizations for image collection in numerous fields. Images captured by UAVs at high altitudes provide a rich source of information on surface topography and terrestrial objects^5,6^. They are indispensable in a range of specialized applications, including environmental monitoring^7^, disaster and emergency response^8^, crop management^9^, urban planning^10^, and in the surveillance of forests and oceans^11–14^, as well as in national defense and security^15^. Marine search and rescue (SAR) represents a notable application in this area. In these scenarios, UAVs capture overhead images of the sea, enabling the accurate identification of objects such as boats, floaters, and life jackets. This information is crucial in enhancing the effectiveness of rescue and recovery operations^16^.

In the field of image object detection, methods utilizing deep learning models have become a hotbed of research, achieving significant strides in this area^17,18^. This encompasses the construction of large-scale, general-purpose image object detection datasets^19–22^ and the development of effective deep learning models^17^. Within these developments, two primary types of models have emerged as mainstream in UAV image object detection: the two-stage models^23,24^, known for their accuracy, such as R-CNN and its variants^25^ (e.g., Faster R-CNN^26^), and the one-stage models^27^, celebrated for their speed, like You Only Look Once (YOLO)^28^. General-purpose image object detection models, as well as those specifically designed for UAV imagery, can be directly adapted or integrated into the marine SAR domain. However, images captured by UAVs for maritime SAR significantly differ from general-purpose images and other remote sensing scenarios^16^. Therefore, a thorough analysis of the unique data characteristics in this field and the development of targeted optimization strategies to improve the accuracy of detection models is of paramount importance for advancing UAV-assisted maritime SAR research.

In response to the aforementioned challenge, our research objective is to enhance the accuracy of two-stage object detection models, known for their precision in UAV-based maritime SAR scenarios, through a data-driven approach that includes the strategic optimization of anchor boxes^29^. By refining anchor box parameters, we aim to leverage their critical role in improving detection efficacy within these complex scenarios. Specifically, we conducted an in-depth analysis and exploration of the data characteristics within the large-scale benchmark dataset SeaDronesSee^16^, tailored for UAV-based maritime SAR. Our focus narrowed down to three particularly challenging categories of objects: swimmers on boats, floaters on boats, and life jackets. This led to the formulation of targeted research questions. Building upon this, we designed an anchor box optimization strategy for two-stage models, employing clustering analysis. The effectiveness of this method was corroborated through experiments, thereby addressing and answering the proposed research questions.

Our contributions are primarily threefold. Firstly, we have conducted an in-depth analysis of the data characteristics in UAV-based maritime SAR object detection tasks, as exemplified by the SeaDronesSee dataset. This analysis identified several object categories that pose significant challenges in terms of accurate detection, thereby formulating the foundation of our research questions. Secondly, in response to these questions, we developed a strategy for anchor box optimization through clustering analysis, aimed at enhancing the detection accuracy of two-stage models in UAV-based maritime SAR tasks. Additionally, we designed and executed experiments to validate the effectiveness of our proposed method. The results and corresponding analyses not only answered our research questions but also deepened our understanding of data in the specialized scenarios.

## Related work

Anchor boxes play a pivotal role in modern object detection models, providing a predefined set of reference frames to facilitate the detection of diverse objects within an image. By encompassing a variety of sizes and aspect ratios, well-defined anchor boxes adeptly adapt to and identify targets of varying shapes and sizes. In the original Faster R-CNN framework as presented by Ren et al.^26^, a combination of anchor boxes with scales of 128, 256, and 512 and aspect ratios of 1:1, 1:2, and 2:1 was implemented, yielding nine distinct initial sizes for these anchor boxes. The Single Shot MultiBox Detector (SSD)^30^ method extends this configuration by including additional ratios of 1:3 and 3:1. Similar approaches are also utilized in other methodologies like Cascade R-CNN^31^, RefineDet^32^, and Guided Anchoring^33^. In these models, anchor boxes are set with common scales and ratios to aid in the detection process. During detection, these predefined-sized anchor boxes undergo localization regression, adjusting in size and position to accurately determine significant areas in the images.

**Figure 1:**
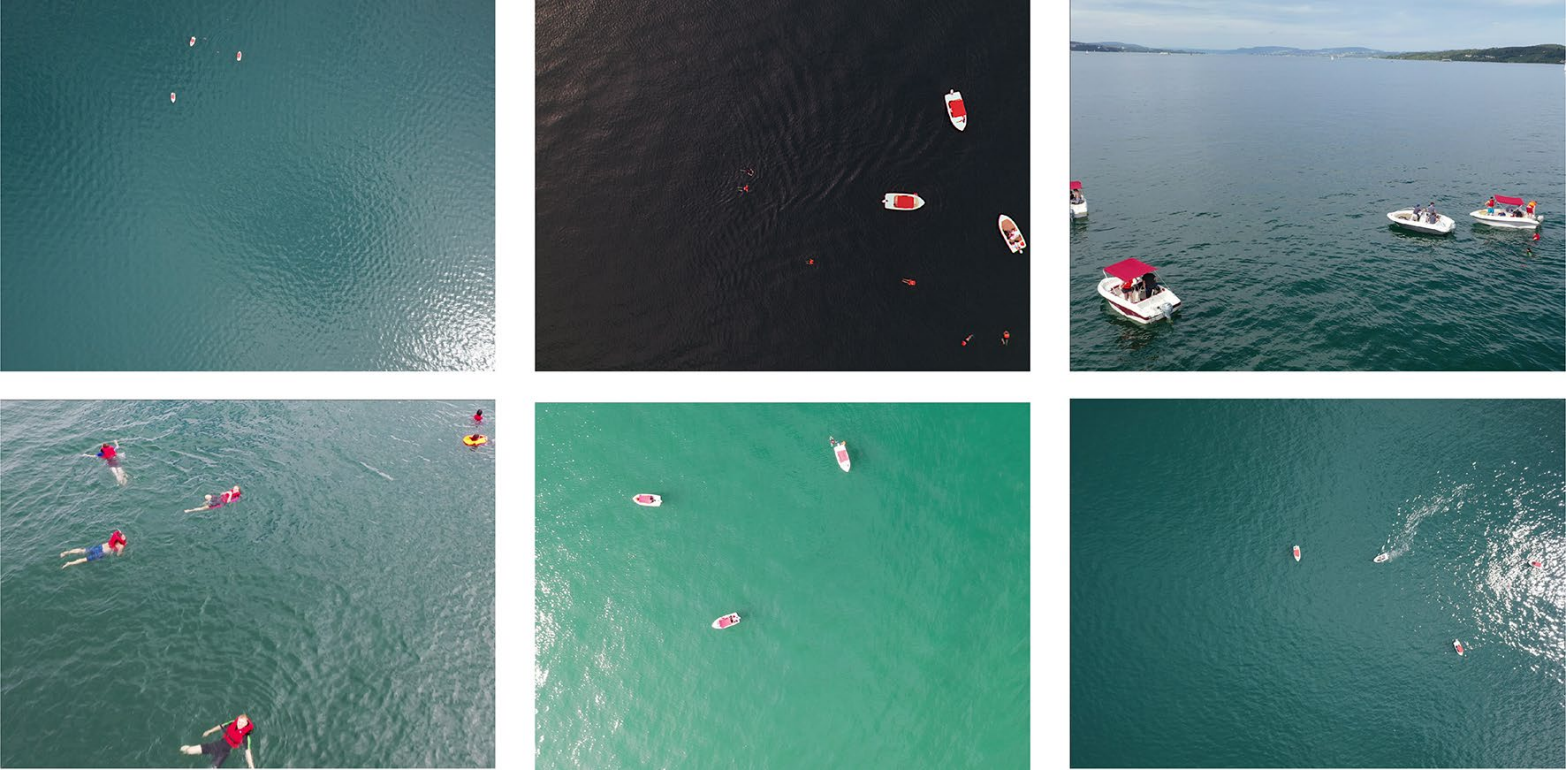
Representative images from the SeaDronesSee dataset, captured by UAVs of varying specifications at different times, altitudes, and angles.

In some studies, researchers have developed adaptive methods to generate optimized anchor boxes. For example, Zhang et al.^34^ designed a semantic-guided anchor adaptive learning network capable of producing anchor boxes optimized for ship targets, thereby enhancing the efficiency of ship instance segmentation models within the hybrid task cascade framework^35^. Yang et al.^36^ and Zhong et al.^29^ approached anchor box dimensions as trainable parameters, enabling the model to dynamically adjust anchor sizes during training based on annotated data. While our method, in contrast to these approaches, relies on clustering analysis of specific datasets and cannot generate anchor boxes adaptively, it is dedicated not only to enhancing the detection precision of the model but also to identifying and investigating objects that are challenging to detect accurately in UAV-based SAR scenarios and tasks represented by specific datasets.

In specialized object detection tasks, optimizing the shape of anchor boxes according to the characteristics of the objects can enhance the detection capabilities of the models. For instance, Liao et al.^37^ proposed additional predefined anchor boxes with ratios of 5:1 and 1:5 for text detection, improving model performance due to the typically elongated rectangular shape of text. Similarly, Najibi et al.^38^ and Zhang et al.^39^ employed anchor boxes with a 1:1 ratio, achieving better results in face detection tasks, as the bounding boxes for faces are generally square. Wei et al.^40^ proposed a clustering analysis method based on Intersection over Union (IoU) to identify more suitable initial anchor box sizes for the COCO dataset^22^. In pedestrian detection, Zhang et al.^41^ observed a substantial impact of anchor box size on model performance and selected an optimized ratio of 0.41 based on data characteristics. Inspired by these data-driven and clustering analysis-based methods of anchor box optimization, our research question emerged, exploring the effect of anchor box optimization on model performance in maritime SAR object detection scenarios using drones, as exemplified by SeaDronesSee, and uncovering the underlying principles.

Building upon the foundational work highlighted above, we observe that while the optimization of anchor box dimensions has been reported to improve model accuracy in various studies^37–40^, there remains a gap in understanding the specific reasons behind these improvements, particularly in relation to the unique data characteristics of different scenarios. In this study, we delve into a detailed analysis of how different anchor box optimization strategies affect the accuracy of object detection in maritime SAR scenarios using drones, such as those exemplified by SeaDronesSee. Through an exploration that combines the analysis of object detection accuracy across different categories with an examination of their visual characteristics, we aim to uncover the sensitivity of various objects to changes in anchor box configurations. This approach allows us to identify object categories that pose significant challenges to detection, thereby revealing the underlying principles that govern the effectiveness of anchor box optimization in maritime SAR scenarios.

## Method

### Data analysis

**Figure 2:**
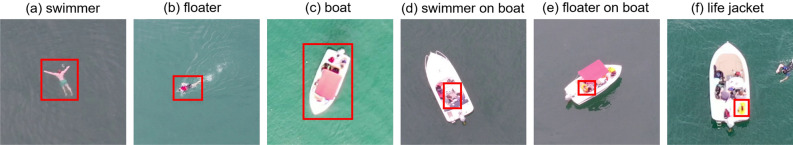
Objects of various categories in the SeaDronesSee dataset, with their annotated ground truth bounding boxes (red rectangles in the image).

We utilized the SeaDronesSee object detection dataset^16^ to analyze data characteristics in UAV-based maritime SAR scenarios, formulating research questions and designing experiments accordingly. Released in 2021, the SeaDronesSee dataset is a large-scale open-water dataset of boats and personnel. This dataset comprises 5630 high-definition images captured by UAVs of varying specifications during search and rescue missions. The images are of high clarity, with resolutions of either 3840$\times$2160 or 5456$\times$3632 pixels. Each object within these images is accurately annotated with ground truth bounding boxes following the COCO standard. Representative images from the SeaDronesSee dataset, along with their bounding boxes, are illustrated in Figs. 1 and 2.

Further, a detailed statistical analysis was performed on the number and area (measured in pixels) of different object categories within the dataset, as illustrated in Table 1. The data reveals a considerable variance in the area of all objects, with the most significant span observed in floaters and boats, ranging from as small as 50 and 165 pixels to as large as several hundred thousand pixels. Even the category with the smallest span, life jackets, ranges from a minimum of 336 pixels to a maximum of 2052 pixels. Based on this observation, we deduce that optimizing anchor box sizes significantly influences the object detection performance in this specific context, particularly for state-of-the-art two-stage object detection models. This effect is further pronounced in models employing the feature pyramid network (FPN)^42^ architecture and complex feature layers. The variations in size among objects of the same category within this dataset underscore the importance of this optimization, highlighting its critical role in enhancing the detection capabilities of such advanced models.

**Table 1:** Statistical annotation information of different object categories in the SeaDronesSee dataset, with areas measured in pixels.

Object	Annotation count	Min area	Max area	Avg area
Swimmer	2480	110	65601	5368.39
Floater	5963	50	172788	6631.17
Boat	7643	165	351330	31416.82
Swimmer on boat	3501	108	35530	4323.04
Floater on boat	1603	100	36736	4361.91
Life jacket	82	336	2052	987.34

Furthermore, we identified two data characteristics that could potentially affect model performance. Firstly, there is an imbalance in the annotation counts across different object categories: the category ’boat’ has the highest number of annotations with over 7000 bounding boxes, while ’life jacket’ has the least, with just over 80. Secondly, there is an overlap among four categories - ’boat’, ’swimmer on boat’, ’floater on boat’, and ’life jacket’, as illustrated in Fig. 2d–f.

Given the analysis presented, the UAV-based maritime SAR scenario data likely pose distinct challenges. These include considerable size variations within the same object category, an imbalance in annotation quantities, and overlapping bounding boxes for certain unique object categories. Addressing these specific characteristics of the dataset is essential, necessitating the development of focused research questions and the exploration of effective methods to resolve and respond to these challenges.

### Research questions

Based on the aforementioned data analysis, we propose the following three research questions that need to be addressed and answered:

- RQ1. Given the overlapping nature of objects such as boats, swimmers on boats, floaters on boats, and the notably scarce annotations of life jackets in the images, are these categories truly challenging to recognize?
- RQ2. The two-stage models have been established as advantageous in terms of recognition accuracy. In UAV-based maritime SAR tasks, can anchor box optimization strategies further enhance the accuracy of such models?
- RQ3. If different anchor box optimization strategies contribute to overall model accuracy improvements, in what specific aspects do these enhancements manifest?

### Proposed method

Overall, we explored the research questions by conducting cross-validation of various models under multiple anchor box optimization strategies. Our methodology is discussed in detail in the following subsections, encompassing four key aspects: model selection, anchor box optimization strategies, validation criteria, and experimental setup.

#### Model selection

For model selection, we employed a series of two-stage object detection models based on the Faster R-CNN framework^26^, combined with different backbones and configurations, including both with and without the feature pyramid network (FPN)^42^. These models have been validated for state-of-the-art accuracy in the field of UAV images object detection^16^. Moreover, anchor boxes play a pivotal role in the object detection mechanism of these models, making them highly suitable for addressing the research questions we have put forth.

Specifically, Faster R-CNN is a two-stage object detection model. The first stage involves a Region Proposal Network (RPN) that generates object proposals. The RPN slides over the feature map obtained from the input image and outputs a set of rectangular object proposals (anchors) along with objectness scores. Mathematically, for a given anchor $a$, the RPN predicts a bounding box regression $\Delta a = (\Delta x, \Delta y, \Delta w, \Delta h)$ to adjust the anchor’s position and size, leading to the refined anchor $a' = a + \Delta a$. The objectness score $o_a$ indicates the likelihood of the anchor containing an object.

The second stage of Faster R-CNN refines these proposals and classifies them. Each proposal is pooled to a fixed-size feature map and then passed through a series of fully connected layers. The output includes a refined bounding box $\Delta b$ and a set of class probabilities $p$. In the context of Faster R-CNN, anchor boxes are predefined bounding boxes of various scales and aspect ratios that serve as references for object detection. The optimization of these anchor boxes is crucial, especially in datasets where objects vary significantly in size.

Integrating the FPN with Faster R-CNN significantly enhances the model’s performance, particularly in the context of anchor box optimization. FPN constructs a top-down architecture with lateral connections, facilitating the use of multi-scale, pyramidal hierarchy features. This multi-level feature representation allows for a more effective deployment of anchor boxes across different scales, compared to a Faster R-CNN model without FPN. In a standard Faster R-CNN without FPN, anchor boxes are applied to a single-scale feature map, which can limit their effectiveness in detecting objects of varying sizes. However, with FPN, anchor boxes are strategically distributed across different levels of the feature pyramid. This approach enables the model to better match anchor boxes with objects of corresponding sizes at different levels of the pyramid, enhancing the model’s ability to detect objects across a broader range of sizes. The combination of optimized anchor boxes and the multi-scale feature representation of FPN provides a robust framework for accurately identifying and localizing objects in complex scenes, making it particularly beneficial for scenarios with diverse object sizes and dimensions.

#### Anchor box optimization strategies

We conducted a comparative analysis of model performances under four distinct anchor box optimization strategies. These included:

1. The default anchor box configuration as provided by a third-party framework (torchvision, in our experiments);
2. The anchor box configuration recommended in the official example code^43^ of the SeaDronesSee dataset;
3. Anchor box sizes derived from IoU-based clustering^40^;
4. Anchor box sizes determined through k-means clustering;
5. Configurations optimized for specific FPN feature map layers based on the k-menas clustering.

Each of these approaches represents a unique method of tuning anchor boxes, offering insights into how different configurations impact the model’s ability to accurately detect and localize objects in marine SAR scenarios.

Specifically, the default configuration in torchvision utilizes anchor boxes with sizes of 32, 64, 128, 256, and 512, and aspect ratios of 0.5, 1.0, and 2.0. In contrast, the official example code^43^ of the SeaDronesSee dataset recommends a broader range of sizes: 8, 16, 32, 64, 128, 256, and 512, with the same aspect ratios of 0.5, 1.0, and 2.0.

IoU-based clustering^40^ optimizes a given list of anchor boxes iteratively. In each iteration, every ground truth box $b_i$ in the training set is assigned to the anchor box $a_j$ in the list with the highest IoU. The distance $d_{ij}(b_i, a_j) = 1 - \text {IoU}(b_i, a_j)$ is calculated, and each ground truth box is assigned to the anchor box $a_j$ for which this distance is minimized, effectively selecting the anchor with the maximum IoU. The average dimensions of all ground truth boxes assigned to each anchor box, denoted as $C_j$, are calculated to determine a more optimal size $\mu _j$. This process is repeated until the sizes of the anchor boxes in the list, now represented by $A^*$, no longer change. The related process is outlined in Algorithm 1, where the set of all ground truth boxes assigned to the j-th anchor box is represented by $C_j$, and the updated list of anchor boxes with optimized dimensions is denoted by $A^*$. We followed the method proposed by Wei et al.^40^ to select a list containing five initial anchor box sizes for iterative optimization. On the SeaDronesSee training dataset, with all images normalized to 1080x1920, the results of the IoU-based clustering optimization for anchor box widths and heights were (10.6, 11.9), (23.4, 28.2), (45.0, 48.3), (60.8, 101.2), and (150.6, 177.1).

**Algorithm 1:**
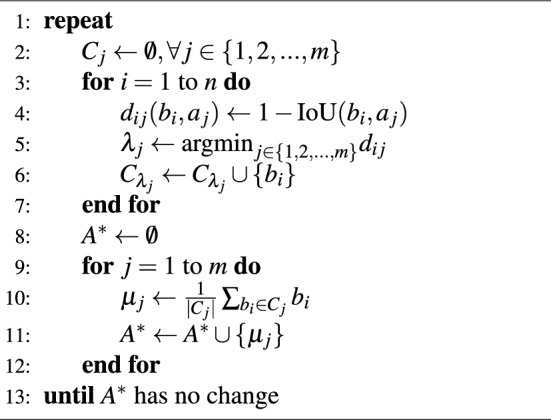
IoU-based clustering method

Regarding the k-means clustering method, we first standardized the input image size to 1080$\times$1920 pixels to address the issue of varying image sizes in the dataset. Following this standardization, an exhaustive analysis of each image’s annotated information was conducted. This analysis involved collecting the widths and heights of all annotated bounding boxes present in the training dataset. Utilizing this data, the k-means clustering method was employed to categorize these dimensions into distinct groups. K-means is an algorithm for partitioning $n$ observations into $k$ clusters, where each observation belongs to the cluster with the nearest mean. It minimizes the within-cluster sum of squares. The mathematical formulation of this objective is:

$$\begin{aligned} \underset{S}{\text {arg min}} \sum _{i=1}^{k} \sum _{\textbf{x} \in S_i} \left\| \textbf{x} - \mathbf {\mu }_i \right\| ^2 \end{aligned}$$

where $\textbf{x}$ represents a data point, $S_i$ is the i-th cluster, and $\mathbf {\mu }_i$ is the centroid of points in $S_i$. By employing k-means on the bounding box dimensions, distinct clusters indicative of common object sizes within the dataset were identified.

**Figure 3:**
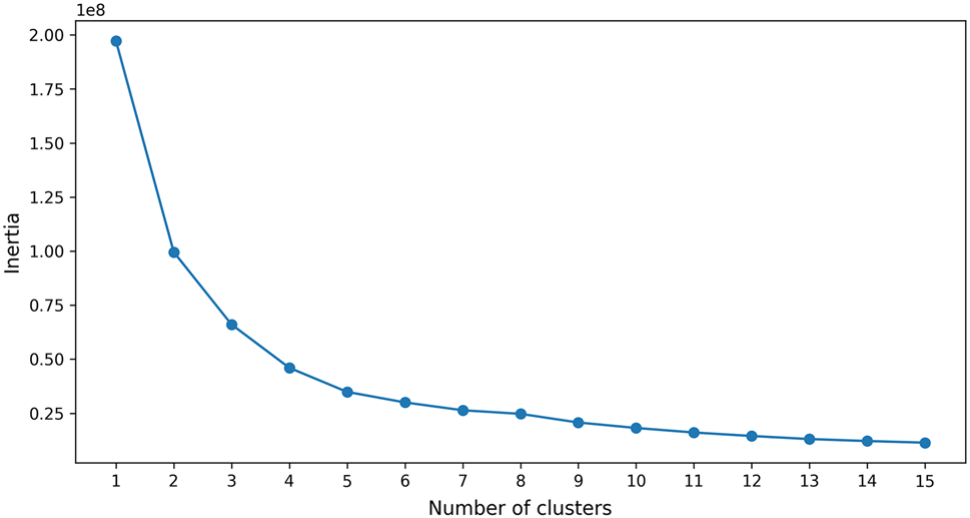
Variation of inertia values with different numbers of clusters using the elbow method.

To determine the appropriate number of clusters $k$, we employed the elbow method which involves calculating and comparing the inertia for different values of $k$ in the k-means clustering process. Inertia, in this context, refers to the sum of the squared distances between each data point and the centroid of its assigned cluster. The values of inertia corresponding to different numbers of clusters $k$ are illustrated in Fig. 3. The principle behind the elbow method is based on observing the rate of decrease in the inertia as the number of clusters $k$ increases. Initially, as $k$ increases, there is a significant reduction in inertia, indicating a substantial gain in defining more distinct clusters. However, beyond a certain point, the reduction in inertia becomes marginal, suggesting limited improvement in cluster definition. The mathematical representation of inertia is given by:

$$\begin{aligned} \text {Inertia} = \sum _{i=1}^{k} \sum _{\textbf{x} \in S_i} \left\| \textbf{x} - \mathbf {\mu }_i \right\| ^2 \end{aligned}$$

where $\textbf{x}$ denotes a data point, $S_i$ represents the set of data points in the $i$-th cluster, and $\mathbf {\mu }_i$ is the centroid of the $i$-th cluster.

**Figure 4:**
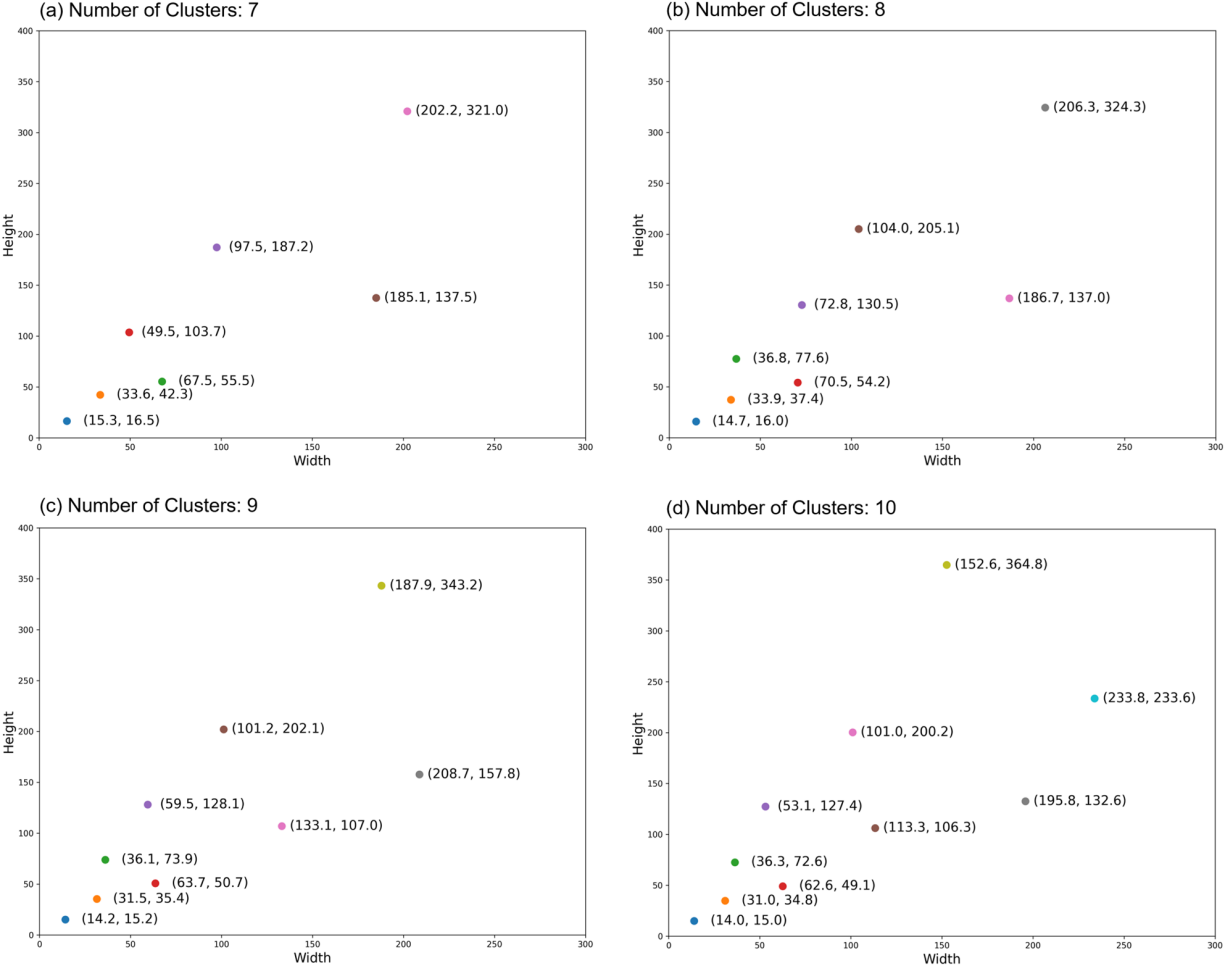
Anchor box sizes obtained from clustering with different cluster numbers. The x-axis and y-axis represent the width and height of the anchor boxes, respectively, measured in pixels. Subfigures (**a**–**d**) correspond to the clustering results with 7, 8, 9, and 10 clusters, respectively.

Based on the elbow method visualization results depicted in Fig. 3, we have preliminarily determined the preferable number of clusters, $k$, to lie between 7 and 10. The visualization of the cluster center dimensions for four different cluster counts, ranging from 7 to 10, is illustrated in Fig. 4. We opted for $k = 9$ as the number of clusters, as this configuration provided a more comprehensive coverage of the two-dimensional space compared to $k = 7$ and 8. For instance, the region around the point (107.0, 133.1) in Fig. 4c is not effectively encompassed by cluster centers in Fig. 4a,b. Furthermore, compared to the case with $k = 10$, the visualization for $k = 9$ does not exhibit any notably vacant areas. Based on these observations, $k = 9$ was selected as the cluster number.

In optimizing anchor boxes for the FPN feature layers, we employed a size-scaling approach grounded in k-means clustering analysis tailored to each specific feature layer. In the case of our experiments with the Faster R-CNN model equipped with an FPN and based on a ResNeXt101 64x4d backbone, its five feature layers were observed to be downscaled by factors of 4, 8, 16, 32, and 64, respectively, relative to the original input image size. Consequently, we scaled the sizes of the anchor boxes fed into each feature layer in accordance with these downscaling factors, ranging from 4 to 64 times smaller than the sizes determined by the cluster analysis.

### Experimental setup and validation criteria

We constructed the object detection model described in Method Section using PyTorch 2.1 and torchvision 0.16^44,45^, and leveraged an RTX 4090 graphics processor to expedite the model training and evaluation processes. Specifically, we crafted distinct Faster R-CNN models, each utilizing ResNet50, ResNet101, and ResNeXt101 64 × 4d as backbones^46–48^, with and without FPN integration. Notably, given the significantly superior performance of the ResNeXt101 64 × 4d backbone compared to ResNet50 and ResNet101, the latter two were exclusively relegated to control groups and were not subjected to further experimentation as backbones for models integrated with FPN. This strategic approach ensures a focused exploration of the most promising configurations, thereby optimizing our investigation of enhanced detection capabilities.

During training, we accumulate four distinct loss components generated by the Faster R-CNN architecture, namely the object loss, RPN box regression loss, classification loss, and ROI box regression loss. These losses are aggregated, and model parameters are optimized through backpropagation. Following each complete epoch of training, the model’s performance is assessed using the COCO evaluation interface^22^, measuring average precision and recall across various Intersection over Union (IoU) thresholds. To mitigate overfitting, we employ an early stopping strategy. Specifically, training halts when the average precision at IoU thresholds ranging from 0.50 to 0.95 (the AP score in Table 2) shows no improvement over ten consecutive epochs. The model with the highest AP score in the training history is then selected as the optimal model.

## Results

### Impact of anchor box sizes on model performance

The evaluation performance of models with various architectures and different anchor box optimization strategies on the SeaDronesSee dataset is presented in Table 2. The models represented in the first eight rows of the table do not employ an FPN, and the results indicate that while transitioning the backbone from ResNet50 to the more complex ResNeXt101 can improve model performance, this enhancement is not as significant as the introduction of an FPN. Moreover, the anchor box optimization strategies based on clustering analysis (whether IoU-based^40^ or k-means) not only failed to improve models without an FPN but also led to negative optimization. The intuitive reason behind this phenomenon is that these models use anchor boxes in a relatively basic manner. Fine-tuning the sizes of the anchor boxes, derived from clustering analysis and lacking extreme small and large values like 2 or 256, without incorporating the FPN’s scaling of image features, actually leads to model deoptimization. The absence of these extreme sizes and the omission of FPN’s image feature scaling compromise regression efficiency, resulting in counterproductive optimization efforts.

In the last five rows of Table 2, the performance of models equipped with an FPN under five different anchor box optimization strategies, as described in the Method Section, is shown. The results clearly demonstrate that anchor box optimization strategies significantly enhance the performance of models with an FPN in the maritime rescue object detection scenario presented by the SeaDronesSee dataset. The two optimization schemes based on clustering analysis not only significantly outperform the default configuration of torchvision but also surpass the anchor box configuration recommended by the official SeaDronesSee sample code^43^. Furthermore, the anchor box configurations optimized layer-by-layer for each level of the FPN, based on clustering analysis, notably exceed the other strategies. In the following subsection, we will delve further into the reasons behind this improvement by analyzing the detection discrepancies across different object categories.

**Table 2:** Performance assessment using COCO metrics: Average precision (AP) and recall (AR), along with category-specific average precision categorized as swimmer (S), floater (F), swimmer on boat ($\text {S}^*$), floater on boat ($\text {F}^*$), boat (B), and life jacket (LJ).

Model	AP	${\text {AP}}_{50}$	${\text {AP}}_{75}$	${\text {AR}}_{1}$	${\text {AR}}_{10}$	S	F	${\text {S}}^*$	${\text {F}}^*$	B	LJ
F.ResNet50-default	11.1	24.8	8.7	6.4	15.6	36.1	28.0	2.4	0.0	82.3	0.0
F.ResNet50-SDS^43^	11.3	24.9	8.5	6.7	16.7	36.5	29.2	2.4	0.2	81.0	0.0
F.ResNet50-IoU^40^	6.3	16.2	4.3	3.8	9.1	20.4	15.1	0.7	0.0	60.8	0.0
F.ResNet50-KMeans	7.6	20.0	4.8	4.6	11.4	25.6	21.5	1.7	0.0	71.4	0.0
F.ResNeXt101-default	13.6	28.4	11.1	7.6	18.3	44.2	37.9	1.6	0.0	86.8	0.0
F.ResNeXt101-SDS^43^	12.7	27.6	9.9	7.2	18.5	41.9	36.3	2.4	0.8	84.1	0.0
F.ResNeXt101-IoU^40^	5.4	13.6	3.9	3.6	8.0	18.6	16.4	0.5	0.0	54.2	0.0
F.ResNeXt101-KMeans	7.4	18.7	5.3	4.4	10.9	23.3	21.9	0.6	0.2	66.2	0.0
F.ResNeXt101-FPN-default	24.9	44.8	22.9	14.0	30.5	71.5	87.3	12.8	0.6	96.8	0.0
F.ResNeXt101-FPN-SDS^43^	32.7	62.8	24.9	22.5	41.8	71.6	88.1	39.2	30.5	96.8	50.5
F.ResNeXt101-FPN-IoU^40^	33.1	67.1	26.4	23.0	42.7	73.7	90.1	37.0	54.2	96.8	50.5
F.ResNeXt101-FPN-KMeans	33.3	66.7	27.3	22.8	41.8	72.0	90.8	43.5	48.1	95.9	50.5
F.ResNeXt101-FPN-FL	36.3	68.8	30.0	25.7	45.1	73.8	90.4	40.5	60.6	96.9	50.5

In model names, “F” denotes Faster R-CNN. Suffixes “-default” and “-SDS” indicate models using torchvision’s default anchor configurations and SeaDronesSee official sample code^43^ recommendations, respectively. “-IoU” and “-KM” correspond to anchor boxes optimized based on IoU^40^ and k-means clustering. “-FL” signifies layer-by-layer anchor size optimization for the FPN layer.

**Figure 5:**
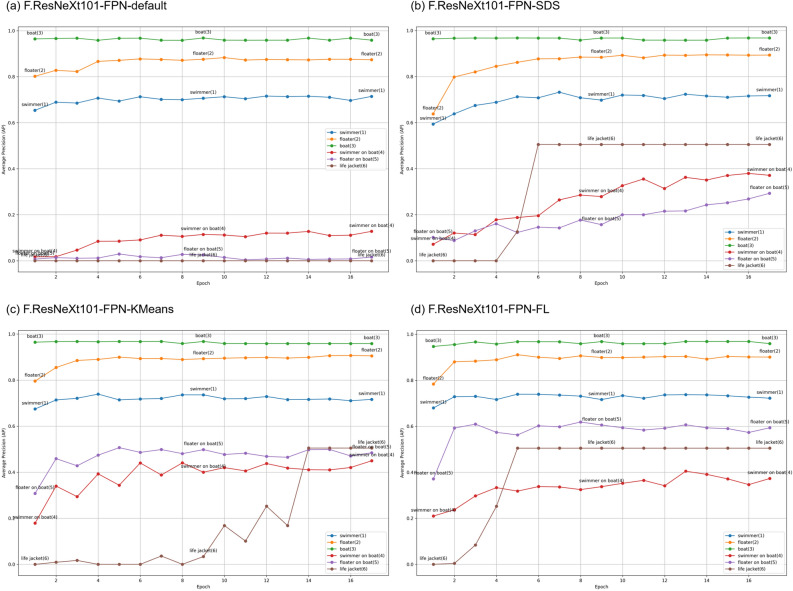
Average detection precision for each category across different anchor box optimization strategies in the FPN-enhanced models during each training epoch.

### Impact of anchor boxes on detecting different categories

From the six columns on the right side of Table 2, it is evident that the model with FPN, even without anchor box optimization, already demonstrates a high degree of accuracy in detecting and recognizing categories such as swimmers, floaters, and boats. The primary reason for the overall improvement in the model’s average precision due to anchor box optimization lies in its enhanced capability to accurately locate and identify three key, smaller-sized object categories that overlap with boats, as outlined in Data Analysis Section, namely, swimmer on boat, floater on boat, and life jacket. To more clearly observe and validate this conclusion, we visualized the average detection precision for each category across different anchor box optimization strategies in the FPN-enhanced models during each training epoch, as illustrated in Fig. 5.

Combining the data from Table 2 and Fig. 5, it is observable that the unoptimized default anchor boxes in torchvision are already highly accurate in detecting common object categories such as swimmers, floaters, and boats. In fact, models optimized with anchor box strategies did not show significant improvement in detecting these standard objects. The main benefit of anchor box optimization is the substantial enhancement in the model’s ability to detect three specific object categories: swimmer on boat, floater on boat, and life jacket. The introduction of more small-sized anchor boxes, denoted as the ’-SDS’ models in the table, improved the detection of life jackets. Meanwhile, anchor boxes optimized through clustering analysis and tailored for FPN feature layers further advanced the model’s capability to identify the more challenging categories of swimmer on boat and floater on boat. Among these, the model further optimized based on cluster analysis for FPN feature layer sizes (denoted as “-FL” in Table 2 and Fig. 5) exhibited significant improvements in recognition across all categories.

## Discussion

### Answers to research questions

Based on data analysis and experimental results, we provide the following answers to the research questions proposed:

- *A1* Among the overlapping object categories, boats are relatively easy to detect, while the other three categories-swimmers on boats, floaters on boats, and life jackets-pose challenges for recognition. The intuitive reason is that, in cases of overlap, boats have a significantly larger area compared to the other three categories, and their bounding boxes encompass the smaller objects with a high-contrast sea background. In contrast, the three smaller categories are much smaller than boats, their bounding boxes are nested within those of boats, and they have a boat texture background that is difficult to distinguish.
- *A2* In UAV-based maritime SAR object detection tasks, anchor box optimization strategies have a modest effect on structurally simpler two-stage models (e.g., models without FPN and with a ResNet50 backbone as used in our experiments), occasionally leading to negative optimization. However, they are highly effective for more complex structured models (e.g., models with an FPN and a ResNeXt101 64 × 4d backbone in our experiments). This effectiveness is evident in various aspects such as the range of anchor box sizes, whether the anchor boxes are optimized based on cluster analysis of bounding boxes, and whether further optimization is tailored for FPN feature layers. The underlying reason is that complex two-stage models are particularly sensitive to the refinement and accuracy of anchor boxes.
- *A3* The effectiveness of anchor box optimization is manifested in the increased detection precision of small, overlapping objects in UAV-based maritime SAR target detection. The more refined and closely aligned the anchor box sizes are with the distribution of actual bounding boxes, the more pronounced this improvement becomes.

### Limitation and extensibility of proposed method

A limitation of the work reported in this article is the scope of validation for our proposed method, which could potentially be applied to a wider range of recent mainstream models. This includes, but is not limited to, Cascade R-CNN, RefineDet, and Guided Anchoring^31–33^. However, constraints in manpower and time limited our ability to extensively validate and test these applications in our experiments. According to the official leaderboard^49^ statistics from SeaDronesSee, the optimized model used in our study has already surpassed many other state-of-the-art approaches. Plans for broader exploration and deeper optimization of these methodologies are part of our future research agenda, aiming to further validate and enhance the applicability of our proposed approach.

The methodology and approaches proposed in this paper hold promising potential for experimental application across various remote sensing or UAV object detection datasets. This includes but is not limited to benchmarks in maritime surveillance such as the SSDD dataset based on remote sensing imaging^50,51^, and UAV-collected datasets exemplified by the VisDrone Dataset^52,53^. Additionally, the anchor box optimization and data analysis methodologies discussed in this paper hold potential for adaptation in other related tasks that rely on anchor boxes. This includes but is not limited to object classification and instance segmentation in maritime remote sensing imagery^54–58^. These applications further underline the versatility and applicability of our proposed methods in a broader context of remote sensing and UAV-based imaging.

In addition, our proposed method relies on analyzing and mining the characteristics of data within specific datasets. Datasets from different scenes and tasks in the real world possess unique features. Our method is anticipated to be applicable in identifying the data characteristics of specific datasets collected by UAVs in the real world, and in optimizing the application of anchor-based object detection models in particular scenes and tasks. For instance, by integrating UAVs commonly used by search and rescue organizations and considering restrictions on flying areas, altitudes, and angles, the data characteristics in these scenarios and tasks can be deeply mined. This information can then be used to optimize and train anchor-based object detection models.

### Discrepancy between validation and test set results

In our experiments, we trained and evaluated models using the officially designated training and validation sets of the SeaDronesSee dataset, which comprise 2976 training images and 859 validation images. The results shown in Table 2 and Fig. 5 originate from this experimental configuration. We observed a decline in model accuracy when applying predictions to the test set, where all models experienced a reduction in performance. For instance, a model scoring an AP of 36.3 on the validation set dropped to 28.9 on the test set (data sourced from the official leaderboard^49^). Potential causes for this reduction might include overfitting control methods, as well as differences in data distribution and annotation quality between the test and validation sets, which may impact the models’ generalization capability. The specific reasons for this phenomenon warrant further investigation in future research. However, given that the performance disparities among models with different anchor box optimization strategies were consistent across both the validation and test sets - that is, models performing better on the validation set also excelled on the test set - our proposed solutions and conclusions regarding anchor box optimization for UAV-based maritime SAR target detection remain valid.

## Conclusions

In this study, we analyzed the detection challenges of objects in a UAV maritime SAR scenario exemplified by the SeaDronesSee dataset. Building on this analysis, an anchor box optimization method was proposed using cluster analysis to enhance the detection accuracy of two-stage object detection models for hard-to-detect objects. Our experiments validated the effectiveness of this approach, particularly highlighting significant improvements in detecting three specific challenging objects in these scenarios: swimmers, floaters, and life jackets overlapping with boats.

The proposed data-driven anchor box optimization method holds potential for application and validation in a broader range of models, datasets, tasks, and real-world scenarios. However, due to constraints in manpower and time, such explorations were not realized in this study. This limitation represents both a shortcoming of the current research and an avenue for future investigation.

## Data Availability

The SeaDronesSee dataset is available at https://macvi.org/. The validation set predictions supporting the experimental results of this paper, which are in the JSON format following the COCO standards, can be accessed at https://pan.baidu.com/s/1zHiIck1_pDn4V3e7gDiJnw?pwd=2024.
